# The Role of Probiotics and Prebiotics in Inducing Gut Immunity

**DOI:** 10.3389/fimmu.2013.00445

**Published:** 2013-12-12

**Authors:** Angélica T. Vieira, Mauro M. Teixeira, Flaviano S. Martins

**Affiliations:** ^1^Immunopharmacology Group, Department of Biochemistry and Immunology, Institute of Biological Sciences, Federal University of Minas Gerais, Belo Horizonte, Brazil; ^2^Department of Microbiology, Institute of Biological Sciences, Federal University of Minas Gerais, Belo Horizonte, Brazil

**Keywords:** prebiotics, probiotics, gut inflammation, microbiota, mucosal immunity

## Abstract

The gut immune system is influenced by many factors, including dietary components and commensal bacteria. Nutrients that affect gut immunity and strategies that restore a healthy gut microbial community by affecting the microbial composition are being developed as new therapeutic approaches to treat several inflammatory diseases. Although probiotics (live microorganisms) and prebiotics (food components) have shown promise as treatments for several diseases in both clinical and animal studies, an understanding of the molecular mechanisms behind the direct and indirect effects on the gut immune response will facilitate better and possibly more efficient therapy for diseases. In this review, we will first describe the concept of prebiotics, probiotics, and symbiotics and cover the most recently well-established scientific findings regarding the direct and indirect mechanisms by which these dietary approaches can influence gut immunity. Emphasis will be placed on the relationship of diet, the microbiota, and the gut immune system. Second, we will highlight recent results from our group, which suggest a new dietary manipulation that includes the use of nutrient products (organic selenium and *Lithothamnium muelleri*) and probiotics (*Saccharomyces boulardii* UFMG 905 and *Bifidobacterium* sp.) that can stimulate and manipulate the gut immune response, inducing intestinal homeostasis. Furthermore, the purpose of this review is to discuss and translate all of this knowledge into therapeutic strategies and into treatment for extra-intestinal compartment pathologies. We will conclude by discussing perspectives and molecular advances regarding the use of prebiotics or probiotics as new therapeutic strategies that manipulate the microbial composition and the gut immune responses of the host.

## Introduction

The gut associated lymphoid tissue is the largest in the body, and mature lymphocytes in the gut mucosa vastly outnumber those in the bone marrow (1). Large amounts of antigens pass through the gut daily, making the gut mucosa the major site of lymphocyte contact with antigens in the entire body. In addition, approximately 100 trillion bacteria are associated with our gastrointestinal tract. This rich gut microbial community referred to as the microbiota has coevolved in a symbiotic relationship with the human intestinal mucosa in such a way that the indigenous microbiota is essential for gut homeostasis (2, 3). The microbiota are considered a “super-organism” and are an integral part of the gastrointestinal tract (4).

Numerous functions are ascribed to the microbiota in the human gut. These partners act similarly to an organ that can provide nutrients and help the host to digest foods, including extraction of additional calories from and metabolism of complex carbohydrates to generate short-chain fatty acids (SCFAs). Furthermore, the gut microbial community is akin to a safeguard of our health because the microbiota compete (for space and nutrients) with potential pathogens and induce the secretion of antimicrobial peptides through interaction with intestinal epithelial cells (5, 6). The gut microbiota can also stimulate the differentiation and proliferation of epithelial cells, which regulate intestinal homeostasis (7–9).

The contributions of the gut microbiota to the development of the immune system have been extensively characterized. There is coordinated cross talk between the gut microbiota and the immune system, allowing the host to tolerate the large amount of antigens present in the gut. Much evidence has highlighted the role of the microbiota in health and disease. The advances in current knowledge of gut microbial biodiversity allow us to understand the mechanisms of how different microorganisms influence host function and these mechanisms’ impact. Altered microbiota (dysbiosis) are associated with gastrointestinal disorders, but more recently, we observed microbial imbalance associated with broad diseases that are not restricted to the gastrointestinal tract (10, 11). The newest evidence shows that the gut microbial composition is associated with malnourishment (which causes one of the highest rates of child mortality in the world) in children from Malawi in southern Africa (12). In this work, Smith and colleagues transplanted the gut microbiota of a malnourished twin with kwashiorkor into germ-free (GF) mice (which are devoid of microbiota) and observed that these animals lost more weight than mice transplanted with the healthy twin’s bacteria. Previously, the association of the gut microbiota with metabolic diseases was demonstrated by the same group, which showed that alterations in the gut microbiota could affect human obesity (13, 14). However, the composition and functional characteristics of a healthy gut microbiota remain to be elucidated.

Strategies that try to restore the normal gut microbiota have been extensively studied in human and animal models, as these methods represent a valuable tool to treat several disorders. A randomized clinical study was the first to report that restoring a healthy gut microbiota is a logical strategy for treating enteric infection (15). The researchers showed the effectiveness of transplanting feces from a healthy patient into patients with recurrent *Clostridium difficile* infections, which can cause severe diarrhea (15). The composition of the gut microbiota varies during childhood, until the individual reaches adulthood. Differences in this composition are related to colonization; host factors, such as sex and age; genetic factors; and health state. The dynamic state of the microbial ecology is increasingly being associated with an expanding number of disorders. However, the importance of perturbations in the gut microbiota and the subsequent impact on the development of inflammation has only recently been recognized. Therefore, ecological principles such as colonization, succession, resistance to change, competition, and cooperation between community members of the microbiota are beginning to be explored (6). In the post-genomic era, new high-throughput methodologies such as metagenomics, transcriptomics, proteomics, and metabolomics have greatly help the understanding the mechanisms by which the microbiota contributes to host physiology in healthy and diseases. Metagenomic studies of the human gut microbiota, for example, have suggested that low bacterial diversity affects host metabolism and is related with obesity and other diseases (4, 16, 17). Both microbial diversity and abundance in the gut are important to maintain human health. The microbiota is essential to prevent the attachment, growth, and penetration of pathogenic microorganisms on the gut surface. The intestinal microbiota play an important role in pathogen resistance, both by direct interaction with pathogenic bacteria and by influencing the immune system (6, 18). The commensal bacteria that reside in the gut are diverse, and in certain cases, individual species appear to have distinct and opposing roles in the gut immune response. The gut microbiota are essential for the development of the immune system. Certain commensal bacteria appear to preferentially drive T-regulatory lymphocyte development, whereas other bacteria promote Th17 development in the gut (19). Much evidence for how the microbiota shape the immune system comes from studies in GF mice, which completely lack microbiota. Such mice exhibit profound immune defects. The development of Peyer’s patches and B and T cell compartments in the lamina propria, spleen, and lymph nodes in GF mice is defective (20–22). Consequently, serum immunoglobulin (Ig) G and intestinal IgA levels in GF mice are reduced (23–25). Additionally, many studies in mice and humans indicate that certain inflammatory diseases are associated with an altered microbiota (26–29). Gut microbiota dysbiosis is related to inflammatory disorders, although whether the microbiota change first, leading to disease development, or respond to another factor is unclear; this is a “chicken and egg” problem. A prospective study of children with a high risk of developing asthma suggested that changes in the microbiota occur before disease development (30). It is becoming clear that certain species of gut commensal microbiota are required for the regulation of immune responses and that perturbations in the microbiota could result in a lack of immune regulation, the outgrowth of more pathogenic microbes, and the promotion of inflammation. The microorganism to which the newborn child is exposed during the first years of life will mainly determine the microbial composition of the microbiota in the adult human gut. Indeed strategies to manipulate the microbiota during infancy have been shown to prevent development of allergic and atopic diseases later in adult life (31–33). Thus, the use of probiotics and prebiotics during the early postnatal period has been proposed to intentionally modulate the microbiota composition. In addition, diet and exposure to microbes during pregnancy may influence the metabolic and immunologic profiles of the pregnant uterus and the risk of disease development in the offspring (34). Thus the administration of probiotics and prebiotics during pregnancy has also been proposed. The influence of probiotics and prebiotics on the gut microbiota in both maternal and infant health has been the subject of recent studies (35, 36).

The rapid growth of metagenomics strategies is being of great help to understand the role of specific microorganism and the overall diversity of the microbiota in many human diseases. This knowledge can help the development of therapies focused on specific effects of different probiotics and prebiotics on the gut microbiota.

## Probiotics and Gut Immunity

The idea that bacteria in the gut could play a role as a regulator of health and disease was first proposed by Elie Metchnikoff more than a century ago. Metchnikoff proposed that toxins produced by a putrefactive microorganism in the colon could inhibit the growth of other bacteria. He came across research noting that a certain rural Eastern European population whose staple was large consumption of fermented milk had unusually long lives. Based on this idea, Metchnikoff proposed that “good” lactic acid-producing bacteria were beneficial to the host by reducing the growth and thus the toxic products of other bacteria within the colon, promoting homeostasis (health) in the host (37). He isolated *Bacillus bulgaricus* and promoted its use as a therapy to maintain homeostasis and prevent aging, thereby popularizing yogurt (37), which formed the foundation for probiotics.

Probiotics are defined by the World Health Organization as “*live microorganisms that can provide benefits to human health when administered in adequate amounts, which confer a beneficial health effect on the host*” (WHO/2001). Clearly, the conceptual basis of probiotics is well grounded. However, the concept that only “live microorganisms” can induce benefits should be discussed. Microbe-associated molecular pattern (MAMP)-elicited signaling has clear effects on epithelial cytoprotection, survival/proliferation pathways, and barrier function (38, 39). Several studies have been show that pattern recognition receptors (PRRs), Toll-like receptors, and Nod-like receptors, have crucial roles in maintaining a healthful stable relationship between the host gut and its microbiota (28, 40). TLR activation results in the up-regulation of pro-inflammatory mediators that facilitate host’s immune defense responses. The NLRs are cytoplasmic proteins that regulate inflammatory responses and activation of these PRRs by commensal microbiota has been evolved to contribute to gut homeostasis (41). However, perturbations of PRR-microbiota interactions, in different cells type and gut mucosal compartment, are more likely to promote disease states associated with exacerbated inflammation (42, 43).

The benefits that probiotics offer to the host have been shown based on data from animal models and clinical evidence, including effectiveness in the treatment or prevention of acute viral gastroenteritis; post-antibiotic-associated diarrhea; certain pediatric allergic disorders; necrotizing enterocolitis; and inflammatory bowel disease (IBD), such as Crohn’s disease and postsurgical pouchitis (Tables 1 and 2). Probiotics have been long reported to aid in the treatment of many dysfunctions of the GI tract, and the mechanisms by which probiotics work have recently been elucidated (Table 3). Clinical trials have shown that the use of probiotics in the prevention of diarrhea can be efficacious especially in newborn and children (44–46). Acute diarrhea is the main symptom of acute gastroenteritis whose most common etiologic agents in children under 1 year are Rotaviruses. There is strong evidence of the clinical benefit of *Lactobacillus* and *Saccharomyces boulardii* treatment to decrease the duration of diarrhea (see Table 2) (45). Diarrhea associated with antibiotics administration is also frequent affecting up to 30% of newborn children (47). Diet supplementation with *Bifidobacterium lactis* and *Streptococcus thermophilus* was shown to reduce the frequency of antibiotic-associated diarrhea (AAD) in infants (48). Increased production of short-chain fatty acids in the colon that stimulates the absorption of sodium by the colonocytes as well as a decrease in intestinal permeability and invasion of pathogenic microorganisms have been proposed to be the major mechanisms by which probiotics reduce diarrheal symptoms (47, 49).

**Table 1:** **Main manufactured probiotic products commercialized worldwide for human applications**.

Product name	Microorganism strain(s)	Manufacturer	Type of product
Probio-Tec^®^	*Bifidobacterium animalis* subsp. *lactis* BB-12	Chr. Hansen A/S, Denmark (http://www.chr-hansen.com/)	Pharmaceutical
Culturelle^®^	*Lactobacillus casei* subsp. *rhamnosus* GG	Valio, Finland (http://www.valio.com/)	Pharmaceutical
Enterogermina^®^	*Bacillus clausii*	Sanofi-Aventis, Italy (http://en.sanofi.com/)	Pharmaceutical
Ultra-levure^®^(Florastor^®^)	*Saccharomyces boulardii*	Biocodex, France (http://www.biocodex.com/)	Pharmaceutical
Miyarisan^®^	*Clostridium butyricum* MIYAIRI 588	Miyarisan Pharmaceutical, Japan (http://www.miyarisan.com/)	Pharmaceutical
Mutaflor^®^	*Escherichia coli* Nissle 1917	Ardeypharm, Germany (http://www.ardeypharm.de/en/)	Pharmaceutical
VSL#3	Four strains of *Lactobacillus* (*L. casei, L. plantarum, L. acidophilus, L. delbrueckii* subsp. *bulgaricus*), three strains of *Bifidobacterium* (*B. longum, B. breve, B. infantis*), and one strain of *Streptococcus salivarius* subsp. *thermophilus*	VSL Pharmaceuticals, Inc., USA (http://www.sigmatau.com/)	Pharmaceutical
Actimel^®^(DanActive)	*Lactobacillus casei* DN-114001	Danone, France (http://www.danone.com/)	Dairy (fermented milk)
Activia^®^	*Bifidobacterium animalis* DN 173 010	Danone, France (http://www.danone.com/)	Dairy (yogurt)
Yakult^®^	*Lactobacillus casei* Shirota	Yakult Honsha Co., Japan (http://www.yakult.co.jp/english/)	Dairy (fermented milk)

**Table 2:** **Main medical/clinical benefits of the major pharmaceutical probiotic products for human purposes**.

Probiotic	Medical/clinical benefits
Probio-Tec^®^^a^	Relieves constipation (50–54)
	Improves fecal properties and microbiota (51)
	Has positive effects against acute diarrhea (52)
	May have an effect on the gastrointestinal system (53)
	Reduces antibiotic-associated diarrhea (54)
	Enhances the intestinal antibody response in formula-fed infants (53)
Culturelle^®^	Prevents rotavirus-related diarrhea in children (55–57)
	Gastrointestinal disorders in childhood (58–60)
	Reduces the risk of respiratory tract infections in children (61–63)
	Useful in the prevention of atopic dermatitis in children at high risk of allergy (64, 65)
Enterogermina^®^	Reduces adverse effects and increases tolerability of *Helicobacter pylori* eradication therapy (66, 67)
	Allergic rhinitis in children (pilot studies) (68)
Florastor^®^	Acute diarrhea (53, 69, 70)
	Recurrent *Clostridium difficile* infection (71, 72)
	Antibiotic-associated diarrhea (73–75)
	Travelers’ diarrhea (76)
	Inflammatory bowel disease (77–79)
	Irritable bowel syndrome (76)
	HIV/AIDS-associated diarrhea (76, 80)
	Reduction of side effects of *H. pylori* treatment (76, 81, 82)
Miyarisan^®^	Antibiotic-associated diarrhea (83)
	Reduction of side effects of *H. pylori* treatment (84)
Mutaflor^®^	Inflammatory bowel disease (85–87)
	Acute diarrhea (88, 89)
	Chronic constipation (90)
	Irritable bowel syndrome (91, 92)
VSL#3^®^	Inflammatory bowel disease (93, 94)
	Pouchitis (95, 96)
	Irritable bowel syndrome (97)

*^a^ Many of the positive effects of BB-12 were observed when it was combined with prebiotic formula and/or other probiotic bacteria, especially *L. rhamnosus* LGG, *L. acidophilus* LA-5, *L. casei* 431, *L. paracasei* subsp. *paracasei* F19, *L. bulgaricus* LBY-27, and *S. thermophilus* STY-31*.

**Table 3:** **Main mechanisms of action of the major pharmaceutical probiotic products for human purposes**.

Probiotic	Mechanisms of action
BB-12^®^	Enhancement of immune response (98, 99)
	Effect on innate immunity (100)
	Modification of microbiota (101)
Culturelle^®^	Prevention of systemic bacteremia (102)
	Improvement of intestinal epithelial homeostasis (102)
	Attenuation of local and systemic inflammatory responses (102, 103)
	Reduction in lipid accumulation (104)
	Secretion of anti-inflammatory substances (105)
	Local induction of reactive oxygen species (ROS) (106)
	Production of bacteriocin (106)
	Interference in bacterium-induced signaling pathways (107)
Enterogermina^®^	Improvement of growth performance and immune response (108)
	Diminishment of intestinal bacterial overgrowth (109)
	Antimicrobial and immuno-modulatory activities (110)
Florastor^®^	Antitoxin effects (111–113)
	Trophic effects on enterocytes (113, 114)
	Anti-inflammatory effects (115, 116)
	Enhancement of immune response (117, 118)
	Enhancement of levels of disaccharidases (119)
	Binding to and elimination of bacterial toxins (120)
	Binding to and elimination of pathogenic bacteria (121, 122)
	Interference in bacterium-induced signaling pathways (123, 124)
	Actions on bacterial virulence factors (125)
	Interference in bacterial motility (126)
	Effects on permeability (127)
Miyarisan^®^	Normalization of intestinal microbiota (128)
	Reduction in substances with harmful effects on intestine (129)
	Antimicrobial effects (130)
	Butyrate production (131)
Mutaflor^®^	Production of colicines (132)
	Adhesin associated to colonization (133)
	Effects on pathogens, epithelial cells, and immune system (134)
	Resistance to colonization by pathogens (135)
	Effects on permeability (136)
VSL#3^®^	Enhancement of immune response/anti-inflammatory effects (137)
	Enhancement of mucosal host defense (138)
	Reinforcement of barrier function (139, 140)
	Production of angiogenesis-promoting growth factors (141)
	Modulation of microbiota (142, 143)
	Reduces inflammation in Food allergy (144, 145)

For the treatment of IBD, several probiotics have been shown to be efficacious, and especially the commercially available mixture VSL#3 (a mixture composed of four strains of lactobacilli: *Lactobacillus casei, Lactobacillus plantarum, Lactobacillus bulgaricus*, and *Lactobacillus acidophilus*; three strains of bifidobacteria: *Bifidobacterium longum, Bifidobacterium breve*, and *Bifidobacterium infantis*; and *S. thermophilus*) (146–148). The *Escherichia coli* strain Nissle 1917 has been demonstrated to improve intestinal homeostasis and to minimize the bacterium-induced reduction of the intestinal barrier, thus decreasing the invasion of intestinal epithelial cells by several pathogens. In this review, we highlight the most commonly used probiotic products and the recently described mechanisms in humans (Tables 1–3). Nevertheless, additional research is still needed to elucidate the functional aspects and the detailed mechanisms of action of probiotics and their impact on human health in relation to various diseases. Different probiotic strains exert their beneficial effects via various different mechanisms and may be synergistic with other microbiota. One probiotic strain may have a different set of properties and clinical effects than another probiotic strain, even if the strains are of the same genus and species. Thus, it is important to note that the efficacy of one probiotic strain does not imply that the other strains will be equally efficacious; rather, further research needs to be performed.

Fukuda and collaborators (149) showed that the probiotic *Bifidobacterium* protects gnotobiotic mice from death induced by enterohemorrhagic *E. coli* O157:H7. However, the protective and probiotic effects were observed only for certain *B. longum* subspecies. In contrast, other bifidobacterial strains, such as *B. longum* subsp. *infantis* JCM 1222T (BT) and *Bifidobacterium adolescentis* JCM 1275T (BA), showed no probiotic effects and failed to prevent the death of mice with O157 infection. The researchers found that this effect can be attributed, at least in part, to increased production of acetate by the protective bifidobacterial strains, which improves the intestinal defense mediated by epithelial cells (150).

Our research group also investigated certain probiotic properties of different microorganisms (*Bifidobacterium animalis* var. *lactis* BB-12, *E. coli* EMO, *L. casei*, and the yeast *S. boulardii*) (118). *In vitro* and *in vivo* tests showed that *B. animalis* and *E. coli* EMO presented better ability to colonize the gastrointestinal tract of GF mice, most likely because the hydrophobic property of the cell walls of these strains increases the strains survival capacity in the gastrointestinal tract of GF mice, without pathological consequences (118). However, *S. boulardii*, followed by *E. coli* EMO and *B. animalis*, induced higher levels of sIgA, and only *S. boulardii* induced significantly higher levels of IL-10 in the intestine. Thus, we concluded that for a probiotic use, *S. boulardii* had better characteristics in terms of immunomodulation in the intestine (118). An investigation of the mechanism by which different probiotic strains trigger a reaction could help to indicate the best microorganism for therapeutic and prophylactic application in several inflammatory diseases. In addition, it is important to highlight that probiotic properties are not only attributed to bacteria. Previous results from our group showed that treatment with yeast *Saccharomyces cerevisiae* strain 905 significantly reduced the translocation and dissemination of pathogenic *Salmonella typhimurium* to all organs in GF and conventional mice after oral infectious challenge (151). The protection conferred by the probiotic against pathogenic bacteria that was observed in this study was most likely due to modulation of both the intestinal and the systemic immunity of mice treated with the probiotic yeast. *S. cerevisiae* 905 induced a reduction in the levels of pro-inflammatory cytokines and modulated the activation of signaling pathways, such as the mitogen-activated protein kinase (p38 and JNK), NF-κB, and AP-1 pathways, which are involved in the transcriptional activation of pro-inflammatory mediators in the intestine (152). In addition, the probiotic treatment elevated the levels of secretory IgA in the intestine and of IgA and IgM in the serum and the production of the anti-inflammatory cytokine IL-10 in the intestine (153). Furthermore, Matins and collaborators demonstrated *in vitro* and *in vivo* that probiotic therapies could be useful as adjuvant when treating gastrointestinal diseases. *S. boulardii, S. cerevisiae* UFMG 905, and *S. cerevisiae* BY474 strains were shown to capture certain pathogenic bacteria on their surface, preventing bacterial adhesion to specific receptors on the intestinal epithelium and subsequent invasion of the host (151, 154). Although most of the conditions under which the useful therapeutic application of probiotics were first described were in the gastrointestinal tract, demonstrating regulation of gut immunity, how probiotics could alter host physiology and function in systemic disorders started to be explored once it became apparent that microbial-immunologic relationships with the host may have implications in extra-intestinal systems.

Although most of the effects of probiotics are beneficial, several negative effects should be considered before therapeutic application. The most important concern about probiotic use is the risk of bacteremia, fungemia, and sepsis (155). Thus, use of probiotics in immuno-compromised patients could generate serious health risks. Probiotic research is moving forward due to basic science and clinical trials evaluating the safety and efficacy of probiotics for various medical conditions. However, clinical trials of probiotics still have limitations and further studies about the amount and interval time are still required cause the benefits of probiotic action vary according to that.

## Prebiotics and Gut Immunity

Prebiotics are broadly defined as a food ingredient that is composed of oligosaccharides that are not digestible by the host and that has a beneficial effect on host health through selective stimulation of the growth and/or activity of specific members of the gut microbiota (156). Currently, only inulin and galacto-oligosaccharides, which are natural food ingredients that are present in certain plants as storage carbohydrates, fulfill all of the criteria for prebiotic classification. Although the previous definition of prebiotics is only applicable to selectively fermented food components, and although much of the prebiotic literature focuses on non-digestible oligosaccharides, most dietary fibers that are fermentable carbohydrates could be considered as prebiotics as well. We hypothesize that any type of dietary or food supplement that could promote the growth of beneficial bacteria and consequently promote homeostasis in the gut and good health could be considered as a prebiotic, even though the supplement may not meet the required criteria.

Fiber carbohydrates (including cellulose, pectin, gums, beta-glucan, and lignin) are not digested in the upper gastrointestinal tract because the host does not have the enzymatic capability to degrade these carbohydrates (157). However, these substances are thought to be selectively ferment by residential bacteria into SCFAs, and particularly acetate, propionate, butyrate, and lactate, once in the colon (158). The vast majority of the bacteria in the colon are strict anaerobes that derive energy from fermentation. The gut microbiota can ferment fiber due to their expression of several enzymes and transport proteins.

Diet alone has the strongest and most direct effects on gut microbial colonization because bacteria have different preferences for different energy sources. Thus, diet is closely related to the species present in the gut microbiota (156, 159). The profile of dominant species in the human gut microbiota can potentially be modified by dietary intake, with consequences for health. The two most abundant phyla found in most healthy individuals are the *Bacteroidetes* and *Firmicutes* (160, 161). Dietary fibers can act as effective prebiotics by inducing major shifts in gut microbial composition and directly affecting the mucosal immune system, resulting in an improvement in enteric inflammatory disorders and the systemic immune response (see Table 4).

**Table 4:** **Main benefits of the major prebiotics and potential mechanisms of action**.

Prebiotic	Medical/clinical benefits	Mechanisms of action	Reference
Inulin	Crohn’s disease	Enhancement of immune response	Emilia et al. (162)
	Colitis	Effect on innate immunity	Macfarlane et al. (163), Ramirez-Farias et al. (164)
	Obesity	Modification of microbiota and increase in *Bifidobacteria*	Hopping et al. (165)
	Diabetes type 2		Costabile et al. (166)
	Colon cancer		Ramnani et al. (167)
	Constipation		
FOS (Fructo-oligosaccharides)	Crohn’s disease	Increase in *Bifidobacteria*	Scholtens et al. (168)
	Colitis,	Decrease in colon pH	Benjamin et al. (169)
	Obesity	Reduction in lipid accumulation	de Luis et al. (170)
	Constipation	Secretion of anti-inflammatory substances	Cummings et al. (171)
	Travelers’ diarrhea	Local induction of reactive oxygen species (ROS)	Arslanoglu et al. (172)
	Colon cancer		Boutron-Ruault et al. (173)
GOS (Galacto-oligosaccharides)	Crohn’s disease	Improvement of growth performance and immune responses	Saavedra and Tschernia (174)
	Colitis	Diminishment of intestinal bacterial overgrowth	Macfarlane et al. (163)
	Obesity		Drakoularakou et al. (175)
Soluble fiber (Guar gum, pectin)	Crohn’s disease	Enhancement of short-chain fatty acid production, and mainly acetate	Peng et al. (176)
	Celiac disease	Normalization of intestinal microbiota	Slavin (177)
	Colitis	Effects on epithelial permeability	Chen et al. (178)
	Colon cancer	Trophic effects on enterocytes	Hu et al. (179)
	Metabolic syndrome	Anti-inflammatory effects	Cao et al. (180)
	Arthritis	Enhancement of immune response	Slavin (181)
	Cardiovascular diseases	Reduction of blood pressure and reduction of LDL serum concentrations	

The anti-inflammatory effects of fiber are likely to be driven by SCFAs. SCFAs have been known to be beneficial for bowel health for many decades. A decrease in “healthy” microbiota and SCFAs is characteristic of patients with IBD, which is most likely due to a reduction in anaerobic bacteria (182). Nevertheless, the delivery of SCFAs has been shown to be successful and effective in reducing colitis (183, 184) and other colonic inflammatory disorders (185). One of the most studied SCFAs is butyrate. Butyrate is the major energy source of colonic epithelial cells affects the proliferation and barrier function of the colonic epithelium and reduces oxidative DNA damage (186, 187). This energy source is transported into cells via monocarboxylate transporters, such as MCT-1107 (188). Butyrate has been shown to reduce the incidence of colon cancer by inhibiting histone deacetylases (HDACs), which affects binding to DNA and thereby transcriptional activity (189). Butyrate, which is more highly produced on a resistant starch, soluble fiber, and inulin diet, was also associated with increased percentages of T-regulatory cells (Treg) and reduced production of IFN-γ, suggesting a down-regulation of inflammation, in an experimental model of IBD (190). High levels of butyrate induced the activation of a nuclear transcription factor and peroxisome proliferator activator receptor γ (PPARγ) (191, 192). PPARγ was first shown to be efficacious in suppressing intestinal inflammation in experimental models of colitis (193). In addition, the activation of PPARγ was shown to reduce pro-inflammatory pathways, such as the STAT, AP-1, and NF-κB pathways, in gut inflammation.

Although most studies on SCFAs have focused on butyrate, acetate is the most abundantly produced SCFA in the colon. Recently, our group and others have shown the anti-inflammatory effects of acetate on the inflammatory response and have started to explore the mechanisms (183, 194, 195). The receptors GPR41 (Ffar1), GPR109A, and GPR43 (Ffar2) were identified as receptors for the SCFAs butyrate, propionate, and acetate (196). GPR41 is primarily expressed by adipose tissue and is also present at very low levels in peripheral blood mononuclear cells (PBMCs). In contrast, GPR43 expression is nearly exclusive to the immune system and is particularly high on polymorphonuclear cells (eosinophils and neutrophils) (183). Our group previously showed that mice that lack the Gpr43 gene have increased inflammation and a poor ability to resolve inflammation because their immune cells cannot bind to SCFAs. Gpr43^−/−^ mice were more susceptible to IBD (183). However, treatment with acetate improved the clinical and inflammatory responses in wt mice, and these effects were dependent on acetate/Gpr43 activation. Interestingly, acetate is largely produced in the colon but reaches a high concentration in the blood, so we could observe systemic anti-inflammatory effects of this SCFA in other diseases, such as asthma and arthritis. More recently, the effect of SCFAs on the specific induction of Foxp3+ IL-10-producing Tregs and the consequent protection of mice against colitis was shown to occur in a GPR43-dependent manner (195).

The molecular mechanism that might explain how a diet enriched in fiber affects the immune system is starting to be elucidated. In addition to increasing the production of SCFAs, such as acetate, propionate, and butyrate, other protective mechanisms of prebiotic activity have been proposed. Prebiotics can also provide resistance to colonization by pathogenic bacteria by inhibiting the adherence of pathogens to the gut epithelium. Other health effects of prebiotics, such as prevention of diarrhea or constipation, positive effects on lipid metabolism, and stimulation of mineral adsorption, are indirectly mediated by regulation of the intestinal microbiota (197).

Our group has demonstrated that another marketed nutritional supplement that has the potential to reduce gastrointestinal inflammation is the marine alga *Lithothamnium muelleri*. Although we still do not know the mechanisms by which *L. muelleri* induces protection in the gut, *L. muelleri* is known to be a good source of polysaccharides and calcium carbonate, which is largely formed in its cell walls. Others species of *Lithothamnium* sp. were shown to be useful as dietary supplements as well and were efficient in chemoprevention of colon polyp formation in animal models (198). These data on the provision of polysaccharides suggest that the use of marine algae as a dietary supplement for anti-inflammatory purposes may provide a novel approach to prebiotics.

Over the past decades, our diet has changed greatly. The consumption of fiber has decreased, accompanied by an increase in high-fat, high-calorie, and processed food. At the same time, the number of people affected by inflammatory diseases has increased. It seems reasonable to suggest that a change in environmental factors, such as diet, in Western societies could be altering our microbiota and therefore our susceptibility to inflammatory disease. In this context, restoring a healthy gut microbiota by external dietary intervention is a logical strategy. Furthermore, given the strong immuno-modulatory function of SCFAs, the production of microbe-derived metabolites using prebiotics is being explored as a promising avenue for prophylactic and therapeutic intervention in gut inflammation. In this way, studies on prebiotics have multiplied in search of a promising new therapy to treat disease and adjuvant therapy along with probiotics, forming symbiotics.

## Perspective on and Future of Symbiotic Therapies

Prebiotics could be administered along with live bacteria (probiotics) that are most able to exploit that energy source to improve the health benefits to the host. The synergistic combinations of probiotics and prebiotics are called symbiotics. The possible health benefits of prebiotics, probiotics, and symbiotics are now being explored in many situations, facilitated by their safety and ease of use. It is important to note that the benefits of regulating the gut microbiota to induce intestinal homeostasis largely extend to other systems in the host. A substantial literature is accumulating on prebiotics and probiotics in several chronic diseases, such as obesity, arthritis, diabetes, cancers, asthma, alcoholism, and cardiovascular diseases, and their effects on host behavior.

The treatment strategies demonstrated by our group using a probiotic *S. cerevisiae* strain enriched with organic selenium in mice and rats with rheumatoid arthritis (RA) resulted in a decrease in several inflammatory parameters, such as infiltration by inflammatory cells, pro-inflammatory cytokine and chemokine production, paw edema, and hypernociception. This inactive yeast enriched with organic Se is a product called Selemax, and we revealed the role of selenium in the amelioration of inflammation associated with the joints. As an antioxidant, selenium plays a vital role in minimizing free-radical activity and is known to strongly influence immune responses. The association of organic selenium and the yeast *S. cerevisiae* (which can transform inorganic Se into organic Se) is a good example of a symbiotic product serving as a promising therapy. Given the effects of prebiotics and probiotics on the gut microbiota, yielding a healthier microbiota and immuno-modulatory properties, and the synergism between these agents, the use of symbiotics to treat many disorders is potentially of great interest. Dissecting the mechanisms by which probiotics, prebiotics, and symbiotics confer benefit on the host will lead to better utilization in treating human diseases involving the immune system.

## Conclusion

Despite recent advances in our understanding of the structure and function of microbial community, we are still only beginning to discover the mechanisms by which changes in the microbiota can affect several disorders. A major challenge to understanding the functional impact of microbial communities on health and disease is the heterogeneity of these communities and the fact that their composition can be influenced by various factors, including host genetics, nutrition, antibiotic treatment, infection, and sequential microbial colonization in the neonatal period. Nevertheless, we have learned that certain species of bacteria can have large effects on the gut immune system and that the balance of these influences is important to the maintenance of homeostasis and the development of novel disease treatment and management strategies. In this context, prebiotics and/or probiotics are a powerful strategy for manipulating the microbial composition and immune responses of the host.

## Conflict of Interest Statement

The authors declare that the research was conducted in the absence of any commercial or financial relationships that could be construed as a potential conflict of interest.
